# A physics-based machine learning technique rapidly reconstructs the wall-shear stress and pressure fields in coronary arteries

**DOI:** 10.3389/fcvm.2023.1221541

**Published:** 2023-09-29

**Authors:** Benjamin Morgan, Amal Roy Murali, George Preston, Yidnekachew Ayele Sima, Luis Alberto Marcelo Chamorro, Christos Bourantas, Ryo Torii, Anthony Mathur, Andreas Baumbach, Marc C. Jacob, Sergey Karabasov, Rob Krams

**Affiliations:** ^1^ Department of Science and Engineering, Queen Mary University of London, London, United Kingdom; ^2^ Laboratoire de Mécanique des Fluides et d’Acoustique UMR5509, INSA Lyon, Ecole Centrale de Lyon, University of Lyon, Ecully, France; ^3^ Bart’s Heart Centre, London, United Kingdom; ^4^ Department of Mechanical Engineering, University College London, London, United Kingdom

**Keywords:** arterial blood flow, shear stress, pressure drop, reduced order modelling, machine learning Frontiers

## Abstract

With the global rise of cardiovascular disease including atherosclerosis, there is a high demand for accurate diagnostic tools that can be used during a short consultation. In view of pathology, abnormal blood flow patterns have been demonstrated to be strong predictors of atherosclerotic lesion incidence, location, progression, and rupture. Prediction of patient-specific blood flow patterns can hence enable fast clinical diagnosis. However, the current state of art for the technique is by employing 3D-imaging-based Computational Fluid Dynamics (CFD). The high computational cost renders these methods impractical. In this work, we present a novel method to expedite the reconstruction of 3D pressure and shear stress fields using a combination of a reduced-order CFD modelling technique together with non-linear regression tools from the Machine Learning (ML) paradigm. Specifically, we develop a proof-of-concept automated pipeline that uses randomised perturbations of an atherosclerotic pig coronary artery to produce a large dataset of unique mesh geometries with variable blood flow. A total of 1,407 geometries were generated from seven reference arteries and were used to simulate blood flow using the CFD solver Abaqus. This CFD dataset was then post-processed using the mesh-domain common-base Proper Orthogonal Decomposition (cPOD) method to obtain Eigen functions and principal coefficients, the latter of which is a product of the individual mesh flow solutions with the POD Eigenvectors. Being a data-reduction method, the POD enables the data to be represented using only the ten most significant modes, which captures cumulatively greater than 95% of variance of flow features due to mesh variations. Next, the node coordinate data of the meshes were embedded in a two-dimensional coordinate system using the t-distributed Stochastic Neighbor Embedding (t-SNE) algorithm. The reduced dataset for t-SNE coordinates and corresponding vector of POD coefficients were then used to train a Random Forest Regressor (RFR) model. The same methodology was applied to both the volumetric pressure solution and the wall shear stress. The predicted pattern of blood pressure, and shear stress in unseen arterial geometries were compared with the ground truth CFD solutions on “unseen” meshes. The new method was able to reliably reproduce the 3D coronary artery haemodynamics in less than 10 s.

## 1. Introduction

Atherosclerosis is the leading cause of death in the developed world, accounting for more than 40% of total mortalities per year. While it has been accepted that risk factors like hypertension, high cholesterol and diabetes play a pivotal role in the progression of the disease, they do not explain the prediliction of atherosclerotic plaque formation near sites of arterial bifurcation, side branching and curvature (1). These predilection sites have been associated with disturbed blood flow and endothelial shear stress patterns (2). Numerous experimental and clinical studies in the last few decades have posited an essential role for disturbed shear stress in initiating atherosclerosis, in progression from simple to advanced plaques, and in rupture of advanced, vulnerable plaques (2). Furthermore, disturbed shear stress patterns are also associated with in-stent restenosis and atherosclerosis (3). Despite the overwhelming number of studies demonstrating the decisive role of blood flow in clinical atherosclerosis, disturbed shear stress patterns have not yet been considered whilst making clinical decisions during catheterization or surgery. This is mainly due to the high computational cost and long convergence times required for sufficiently accurate numerical solutions. Several propositions have been made to reduce time requirements, of which one of the earliest attempts was by applying supercomputers to the numerical solvers (4). While this reduced convergence time from a full day to a few hours, a condition now met by standard modern computers, this is still not sufficient to aid in diagnostics. Clinical decisions depend on data which can be reliably obtained within minutes, preferably seconds. Hence, newer statistical modelling methods were used to further reduce convergence time of Computational Fluid Dynamics (CFD) simulations based on machine learning (5). These can roughly be divided into two categories, the classical machine learning methods and physics-based machine learning methods. Classical machine learning methods use the power of deep learning to estimate wall shear stress profiles (5). The advantage of these methods is the flexibility of the feature space to predict these wall shear profiles primarily due to the high expressivity of Deep Neural Networks (DNN) and their ability to identify high dimensional features. However, such methods are not based on capturing the inherent physical conservation laws of the governing fluid flow. Consequently, any change in feature space will necessitate a DNN recalibration cycle.

To overcome the above, physics-based machine learning technologies have raised interest recently. These methods are predicated on capturing the underlying physics either via incorporation of the actual conservation laws (6) or by data-driven extraction of physically interpretable flow characteristics (7) as features for regression. For instance, Reduced-order modelling of CFD simulations are motivated by the presence of coherent structures, identified from their statistical moments in the datasets available from short duration simulations (8, 9). By applying orthogonal decomposition theory, it is possible to identify high energy Eigenvectors, also known as modes, of these coherent structures using essential information of the flow solution field (e.g. 3D velocity and pressure) while reducing dimensionality of the data. Initial studies used both temporal and spatial information of the velocity field to reduce its dimensions in non-health related areas (7). The first health applications used these methods to study coherent structures in the velocity field of idealised phantoms of bifurcations, saccular and aortic aneurysm (10). Patient-specific applications, which are noisier, have been successfully studied by accounting for such noise in the signal (11). In order to apply these reduced order flow solution fields to novel objects, an interpolation needs to be carried out.

In light of these advances in closely related fields of research, this paper establishes the foundation of our novel method amalgamating these techniques and applies it to a well-characterised experimental dataset of atherosclerotic pig coronary arteries (12). We will show how to modify classical POD, introduce a shape optimizer for blood vessels, and present a suitable Random Forest Regressor (RFR) model to predict flow fields in novel arteries.

## 2. Outline of methodology

**Figure 1:**
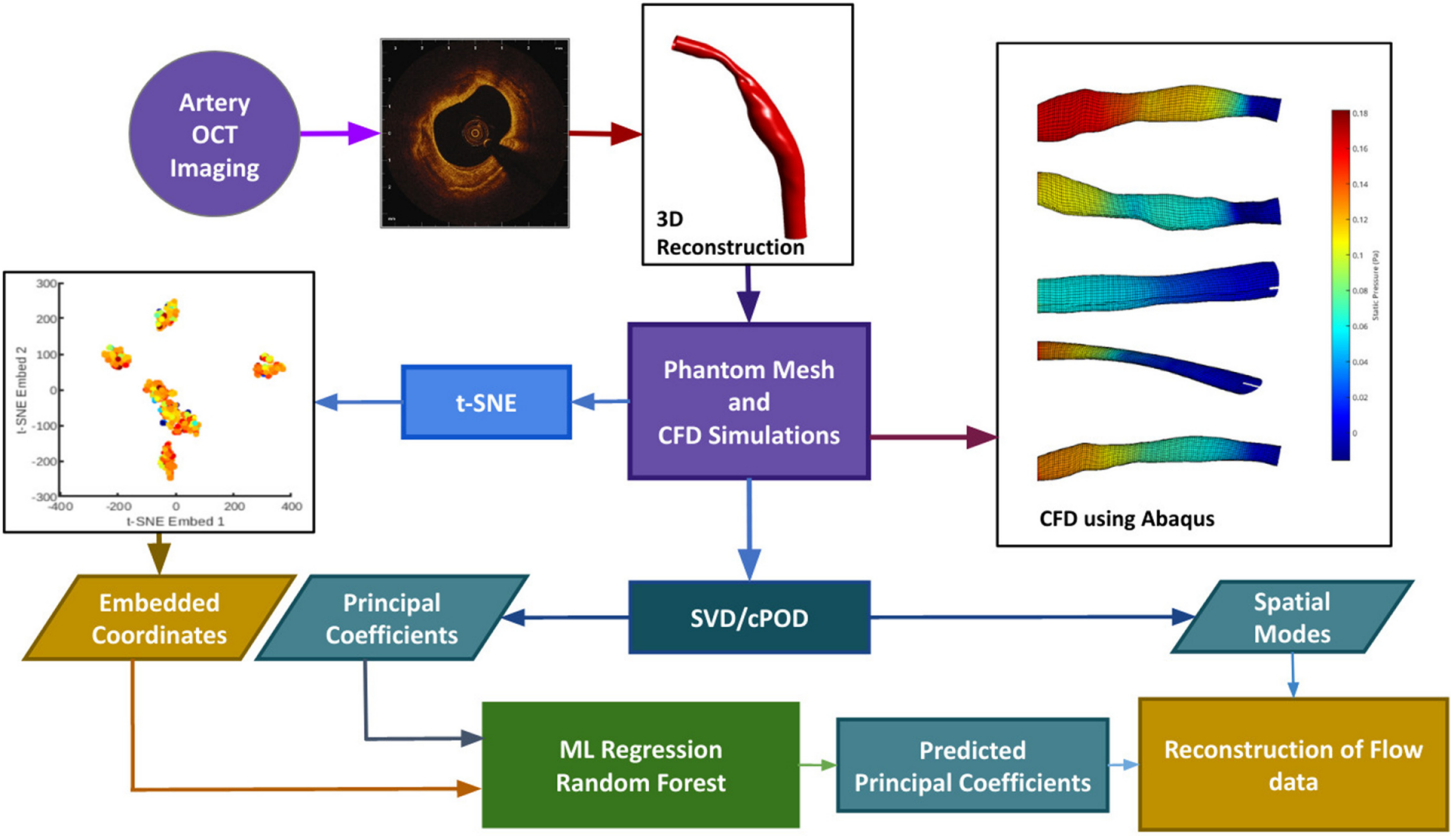
The data processing pipeline is summarized in this flowchart. OCT images are obtained in the cath. lab. and used to extrapolate a 3D contour. Mesh generation and Computational Fluid Dynamics are done through an automatic pipeline. The velocity profiles obtained from CFD will act as the ground truth. Synthetic data generation (*n* = 1407) is done by random purturbation of the length-wise diameter of each independent blood vessel (*n* = 7). Data reduction is performed on the shear stress, and pressure fields obtained from CFD, via POD (see text for details), and on the input meshes through t-SNE (see text for details). These reduced data sets are used to train (using 90% of the data) and validate (using 10% of data) the machine learning learning module

We have developed an automatic pipeline which generates synthetic data from existing 3D reconstructed blood vessels (12), performs proper orthogonal decomposition (POD) on the shear stress and pressure field solutions, and t-distributed Stochastic Neighbour Embedding (t-SNE) on the mesh coordinate data to enable feature reduction. The reduced mesh and flow parameter fields are then used to train, validate and test a RFR model to perform interpolation; thereby enabling a fast reconstruction of CFD solution in a given geometry. In the case of an unseen geometry as test input, the position of the corresponding geometry in the t-SNE space is calculated analytically, and the mode coefficients are predicted using RFR. Recombination of the previously extracted mesh-wise modes along with the newly predicted POD mode coefficients is then used to produce the flow field solutions for the new geometry. The pipeline is summarised in the form of a flowchart as shown in Figure 1, and the methods are described in Sections 3, 4, 5 and 6.

## 3. Creating a well-annotated synthetic data repository

**Figure 2:**
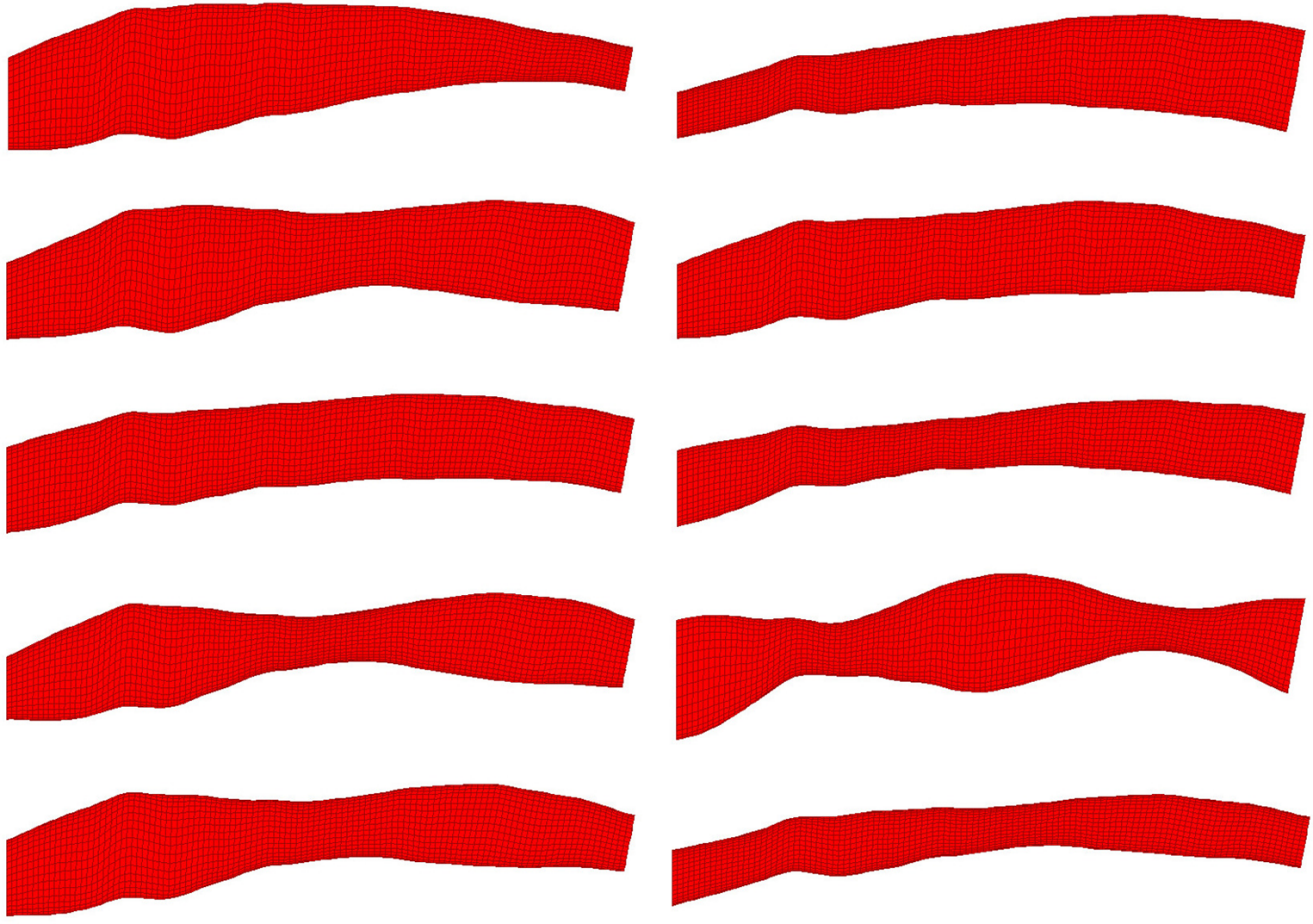
10 randomly selected phantom geometries from the dataset are visualised. All phantoms shown were generated from the same OCT image. Variation in shape is due to random synthetic perturbations applied to the artery diameter, the function of which is a composite of two sinusoids with randomised amplitude, frequency, phase and vertical displacement. This ensures smooth, continuous variation along the length of the artery regardless of input parameters.

Synthetic data has been proposed to meet the huge data requirement of artificial intelligence (AI) (13). Here, we developed a hybrid technique which uses a combination of realistic and synthetic data. The realistic data was obtained from a validated 3D reconstruction method of coronary arteries based upon a pullback of OCT images and angiography (Figure 2). This 3D vessel anatomy was then used as a seed to generate synthetic data by applying random spatial perturbations to the original mesh. To prevent unnatural, discontinuous geometric differences within each mesh phantom, the perturbations are based on the amplitude of a sinusoid, which distributed the perturbation lengthwise. The sinusoid components have independently randomised amplitude, frequency, phase and vertical offset. With this method, 200 phantom meshes per each of the 7 unique blood vessels available were generated. Including the 7 natural artery shapes, this results in a total of 1407 3D meshes in this preliminary dataset. These geometries were then input to the CFD solver Abaqus (v16.2) to obtain the pressure and shear stress field by solving the governing steady-state incompressible Navier-Stokes equations. In the solver, the governing equations were discretised on ∼100,000 mixed hexahedral and triangular prismatic elements in accordance with the second order of approximation. The advection term in the momentum equation was discretised using second-order least squares. To accelerate convergence of the steady solution with imposing the divergence free velocity field, the pressure-correction method (SIMPLE) was used with an efficient solution of the Poisson pressure equation. Boundary conditions were imposed as constant inflow (100 cm/s), and zero pressure outflow. On all vessel walls, a zero velocity and logarithmic wall function boundary condition was specified. Blood rheology was modelled as a non-Newtonian fluid following the Carreau-Yasuda model, which at high strain rates incorporates the effect of shear thinning in the definition of kinematic viscosity as:

$$\nu =\nu_{\infty }+(\nu_{0}-\nu_{\infty })(1+(\tau \dot{\gamma }{)}^{\alpha }{)}^{\left(n-1\right)/\alpha },$$

where $\dot{\gamma }=\partial u/\partial y$ is the flow shear gradient near the wall, and the model coefficients are summarised in Table 1. For turbulence modelling, the standard k– ε RANS (Reynolds Averaged Navier Stokes) model was used. All calculations were performed using APOCRITA, the HPC cluster of Queen Mary University of London (14).

**Table 1:** Parameters of the Carreau-Yasuda model.

$\nu_{\infty }$, $m^{2}/s\times 10^{6}$	$\nu_{0}$, $m^{2}/s\times 10^{6}$	τ, s	α	n
3.45	56	3.313	2	0.3568

## 4. Data reduction of the CFD solution fields using proper orthogonal decomposition

POD is a tool in CFD post processing and is derived from the Singular Value Decomposition (SVD) method for matrix factorisation commonly used in statistical analysis. The method finds correlations in the vector flow solution field, which contains small linear perturbations, to obtain an Eigenbasis onto which the mesh flow data can be projected. In classical POD, the correlations are obtained in the time domain to identify flow structures that are most dynamically important in time during the evolution of turbulence. The same methodology is also extended to varying flow cases based on different experimental setups (e.g. considering a number of unsteady flow experiments performed on the same CFD mesh), this is known as common base POD (cPOD) (15). In our methodology for obtaining common mode functions underlying multiple meshes, the time domain is replaced with the domain of the mesh geometries. It is assumed that a few smoothly varying variables can be used to represent the mesh cases. The goal is to obtain the hidden common modes in the stationary solutions, on multiple meshes, while the mesh is smoothly varied. To obtain the modes underlying the variations in pressure and shear stress fields, we use the method of SVD. We begin with a dataset of CFD simulated steady-state flow solutions. For one simulation, the chosen output variable (e.g. pressure and wall shear) is organised into a N-length vector, where N is the number of nodes in the mesh. These vectors are oriented horizontally and then stacked vertically. With M meshes, the resulting 2D solution matrix A has the dimensions M×N. Our application of SVD follows the theory of snapshots (16), similar to other use cases. However, each snapshot (stacked vector) in our solution matrix is not a different time frame of the same simulation, but rather a steady state solution ran with identical conditions on a different, uniquely shaped mesh. SVD factors the matrix into a product of three matrices $A=UDV^{T}$, where the columns of U and V are orthonormal (V is transpose), and the singular matrix D is diagonal with positive real numbers, organised by magnitude in descending order. The sum of the singular values represents the total amount of information in the system. They are analogous to the Eigenvalues of the Eigen decomposition, and represent the magnitude, or significance, of each Eigenvector, or POD mode. The singular values can then be used to estimate the number of modes needed to reconstruct the flow solutions without significant loss of information (16). Both vector matrices U and V are organised in terms of the singular values, from most to least significant. The summed energy of each leading mode, being their corresponding singular values, are then used to define a tolerance threshold for information loss. Due to spatial coherence of particular modes of variation of the flow with respect to the mesh shape, the number of modes that capture the majority of useful information are the first few, as compared to the full dataset. Modes that fall outside of a chosen threshold in terms of correlative significance can be truncated from the dataset, drastically reducing the dimensionality of the data whilst incurring a tolerable underestimation of the concerned node-wise flow parameter. Additionally, although not implemented in the current case, explicit smoothing can also be applied in the correlation matrix space to enhance numerical properties of the meh-wise POD coefficients (8). In this case, the leading 10 modes were found to capture >95% of total information about both the pressure and wall shear stress, and thus were deemed sufficient for accurate reconstruction.

## 5. Data reduction of the synthetic meshes of coronary blood vessels

Several shape optimizers have been proposed in the literature, of which t-SNE has acquired a lot of attention (17). The t-SNE is a statistical method for visualising high-dimensional data by embedding each N-dimensional data point in a reduced space, typically of two or three dimensions. A higher number of embedding dimensions will retain a greater accuracy of clustering, but also increase the sparsity of data within the space. More specifically, t-SNE generates the joint Gaussian distribution of the conditional chance that a nearby mesh coordinate is sufficiently close in terms of Euclidean distance to an initial mesh coordinate. The unknown variance of the Gaussian distribution is obtained from the Shannon entropy. This step creates a matrix of each mesh coordinate with all other mesh coordinates where a chance is provided on the basis of distance.

As a next step, a reduced order mapping is obtained by minimizing the Kullback-Leibler divergence between the Gaussian distribution of the original points and a Student’s t-distribution of points in a reduced dimensional space. The resulting vectors are then used to fill the feature space. In a sense, the space is “seeded” with the meshes produced from the natural OCT images. The space around each image is then populated with the synthetic mesh vectors, which have a small but significant geometrical difference from the parent mesh. The goal being to fill the feature space and bridge the empty regions between the clusters. Given that the principal coefficients are physics-based, they should maintain a causal link to the values of the embedding coordinates, which represent variability in mesh shape. A filled feature space with an intact causal link will aid an interpolative machine learning model to make accurate coefficient predictions for an unseen geometry (Figures 5 and 6). It is worth noting that what constitutes a “filled” feature space is highly dependent on the chosen t-SNE parameters and the natural limits of the data that is being reduced. The “natural limit” is in reference to the fact that a hypothetical dataset containing all possible natural variations of the artery shape will produce a “filled” feature space, and the regions that are not populated will represent shapes that do not occur naturally, and thus may not be useful for a diagnostic tool. Hence, we aim to produce synthetic data, which is not so different from the natural data as to have its shape fall outside of this hypothetical set. It is for this same reason that it is better to bolster the dataset with natural shapes wherever possible, with synthetic data playing a supplemental role. Integration of human OCT patient data is forthcoming in future research.

## 6. Random forest regressor and regressor chain

SVD re-organizes the modes based on their energy level content and the number of modes are truncated when >95% of the variance of the field is preserved. This resulted in the first 10 modes for the pressure field and the shear stress field for the dataset we use for this study, which when used for reconstructing the solution leads to a root mean squared error less than 5%. In order to interpolate the POD principal coefficient field that enables predictions of future objects, simple feed-forward neural networks and classical machine learning methods were compared. It was found that the RFR algorithm combined with the Regressor Chain algorithm were best suited for this task.

The RFR algorithm is a supervised machine learning technique that integrates multiple independent decision trees on a training data set: the obtained results are ensembled to obtain a more robust single model compared to the results of each tree separately (18). RFR is a supervised learning method in the sense that during training it identifies mappings between inputs and outputs. In our setup, the t-SNE coordinates of the meshes are the input and the cPOD principal coefficients are the output. In our approach, an independent RFR regressor is employed for each of the 10 coefficients. The Random Forest Regression algorithm utilised in our work is obtained from the popular Machine Learning library Scikit-learn. Scikit-learn is built to facilitate the use of Artificial Intelligence and Machine Learning algorithms, and is used in regression, classification, and clustering tasks. The model is imported as “sklearn.ensemble.RandomForestRegressor.” Additionally, a Regressor Chain architecture is used to obtain a multiple output model that organises the regression of individual modes in a chained fashion. Thus, RFR creates a regression model for each pressure coefficient, where each model makes a prediction for its coefficient specified by the chain by using all the t-SNE features provided to the model and the predictions of previous outputs in the chain. This ensures that the correlation between the features are taken into account to enhance the regression.

## 7. Results

**Figure 3:**
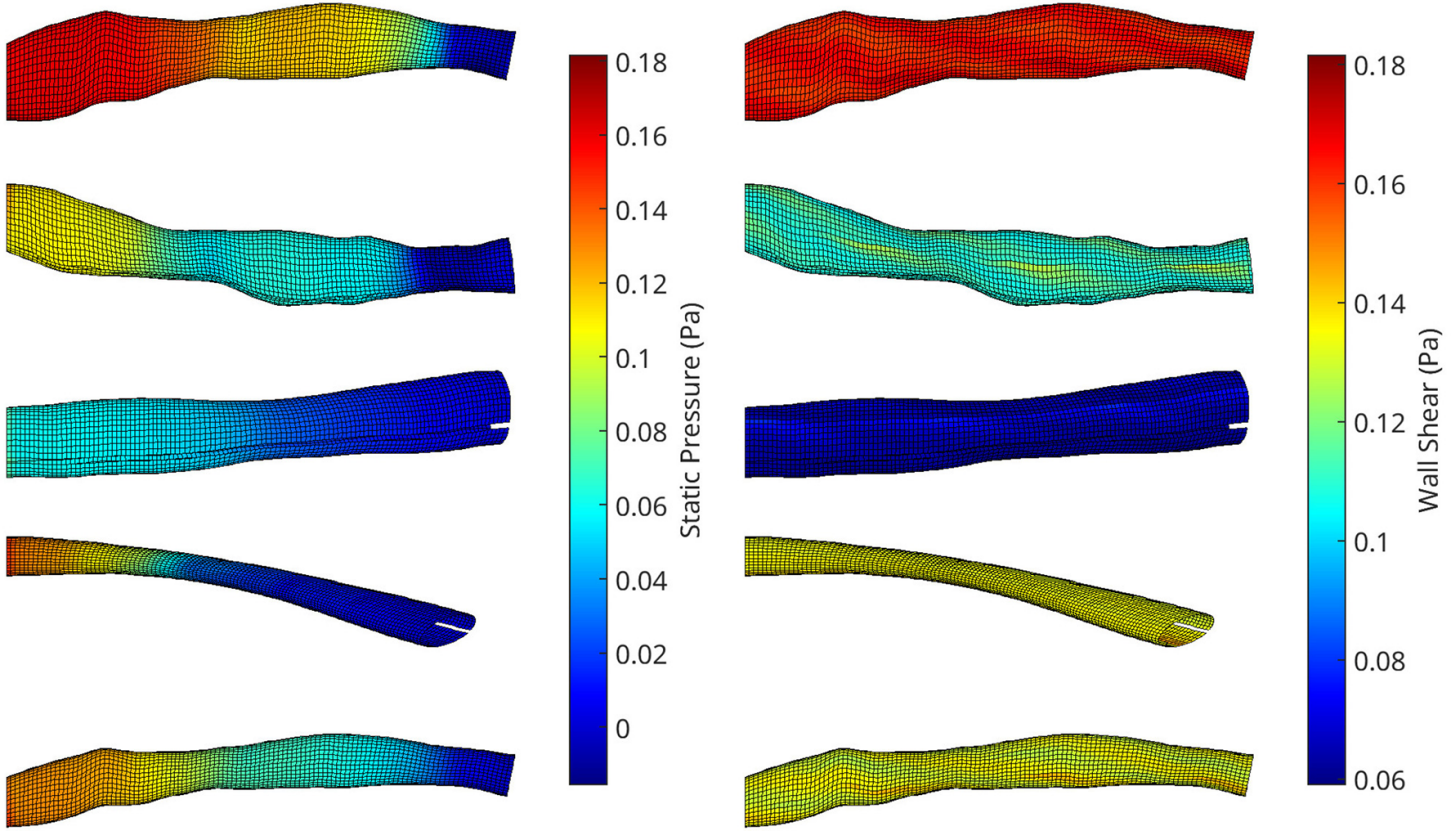
A collection of meshes generated using various OCT images and perturbation parameters, coloured by the pressure (left) and wall shear (right) solutions from CFD simulations. The mesh dimensions are normalised for the sake of visualisation.

**Figure 4:**
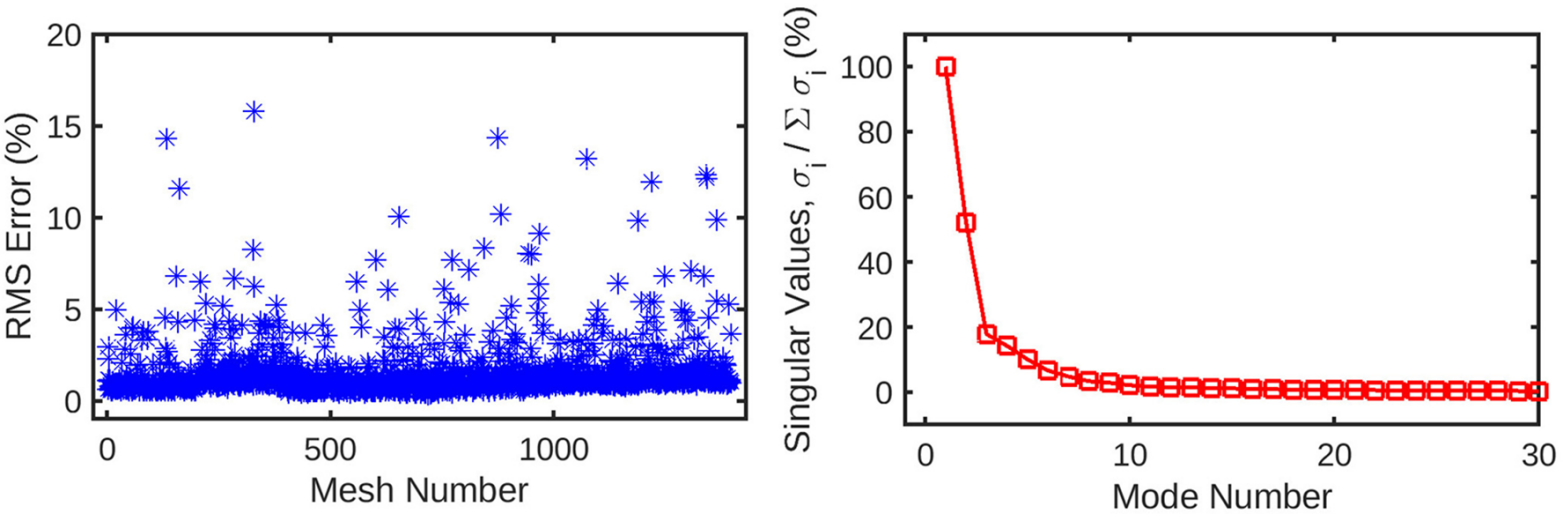
(left) Root-mean-squared error for the reconstruction of the original mesh-wise pressure solution from a truncated set of 10 principal coefficients per mesh. The error is normalised against the range of pressure values across all meshes. (right) Singular values for the decomposition of the pressure solution, normalised against the largest value. These singular values are ordered by magnitude and represent the relative contribution of each POD mode to the energy of the overall pressure solution. Subsequent values quickly decay to <1% of the highest value, as the first several modes represent the overwhelming majority of the information in the pressure field. This indicates that many of these trailing modes can be safely discarded from the dataset without losing a significant amount of information.

**Figure 5:**
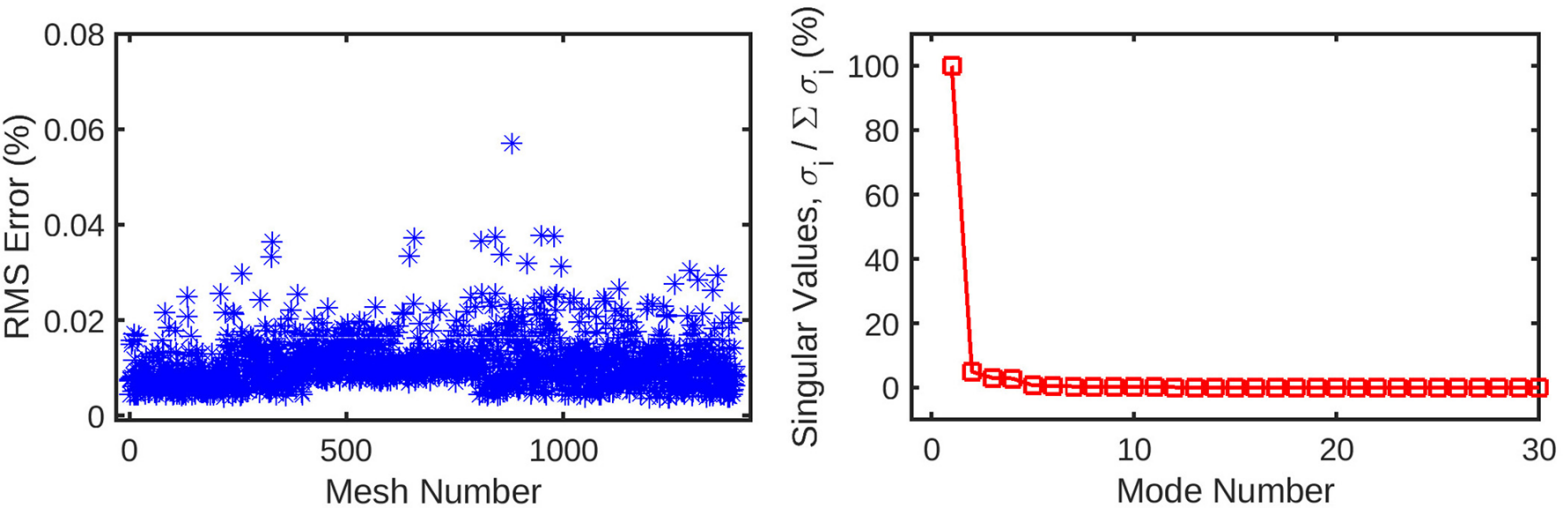
The mesh-wise reconstruction error for wall shear (left) is much lower than pressure reconstruction using the same number of coefficients. Additionally, the singular values (right) decay to 0 in a fewer number of modes compared to the pressure decomposition. These factors are indicative of the wall shear solution being easier for the POD method to decompose than static pressure, possibly due to the fewer number of CFD nodes for which it is computed.

An automatic pipeline was implemented to perform highly accurate 3D reconstruction from biplane angiograms and an OCT pullback (19), to automatically generate a mesh and on basis thereof, and to generate small perturbations in the topology of meshes. The latter was then used to generate a full stationary solution of the shear stress and pressure fields using the Navier-Stokes solver in Abaqus. The perturbation parameters were bounded to induce small but significant changes in the accompanying geometry of the meshes (Figure 2). This also resulted in appreciable changes to the pressure and wall shear fields (Figure 3). The cumulative wall shear stress and pressure fields were then further analysed with the cPOD procedure. The first 10 modes of the pressure and shear stress fields were sufficient to reproduce >95% of the variance of both fields, leading to modest errors in the reproduction of the original fields of <1% (Figures 4 and 5).

**Figure 6:**
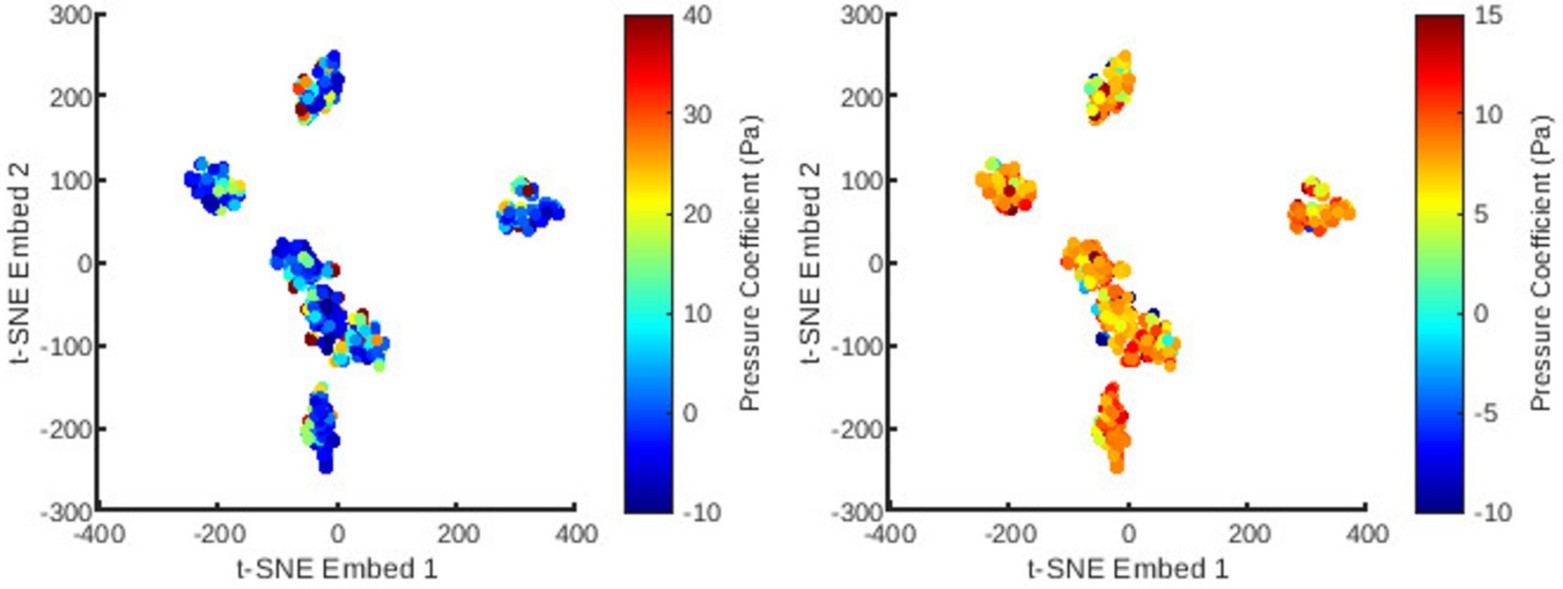
The distribution of all meshes in the database embedded in 2D t-SNE space with colours representing the principal coefficients of the static pressure solutions for the first (left) and second (right) mesh wise POD modes.

**Figure 7:**
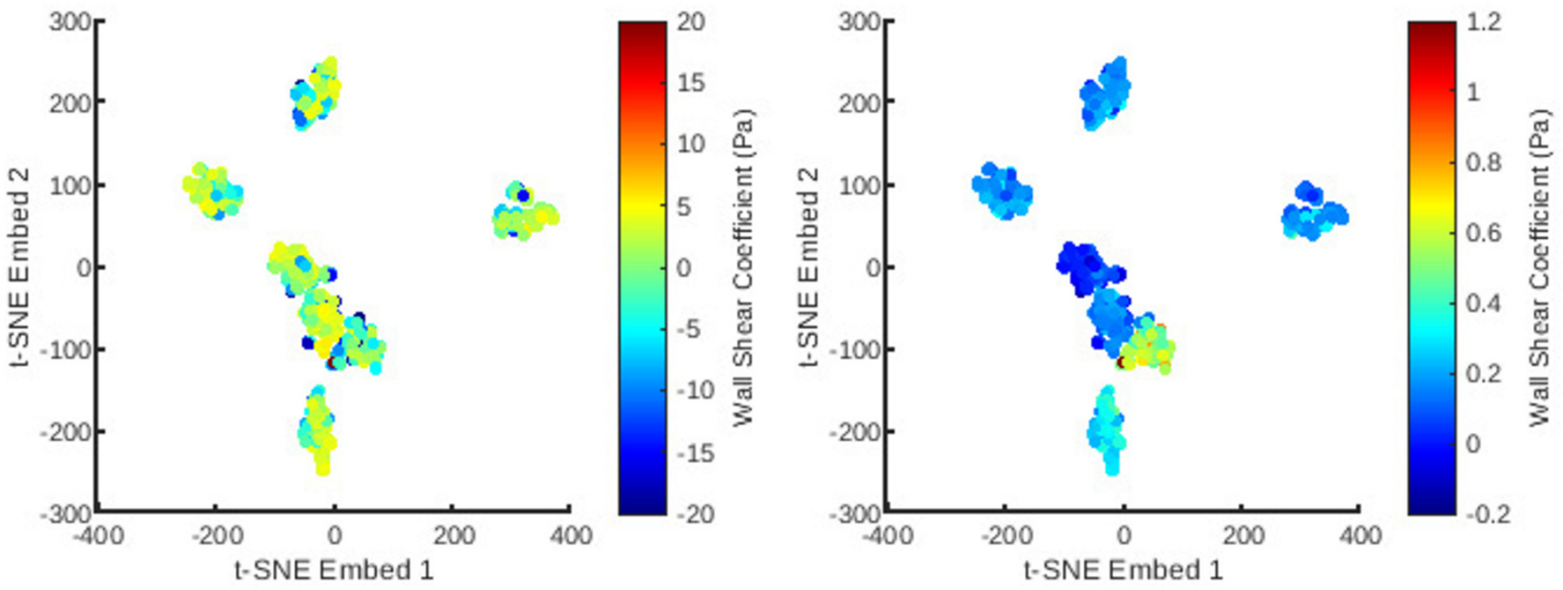
The distribution of all meshes in the database embedded in 2D t-SNE space with colours representing the principal coefficients of the wall shear solutions for the first (left) and second (right) mesh wise POD modes.

Next was a reduction in the dimensions of the mesh topology using t-SNE (Figures 6 and 7) for utilisation in a low-dimensional regression task. The t-SNE algorithm enables control over the clustering behaviour based on similarity through its perplexity parameter. This was fine tuned to obtain an approximately homogeneous distribution of the mesh cases, whilst preserving noticeable clustering features. This allows for a smooth geometrical representation suitable for regression. As can be observed, the t-SNE features resolve to seven clusters corresponding to seven natural artery shapes. To which, random perturbations are introduced to generate quantitatively distinct synthetic datapoints. Additionally, within each of the t-SNE clusters, the variation of the principal coefficients are also smooth and continuous since their values are correlated with variation in mesh shape.

**Figure 8:**
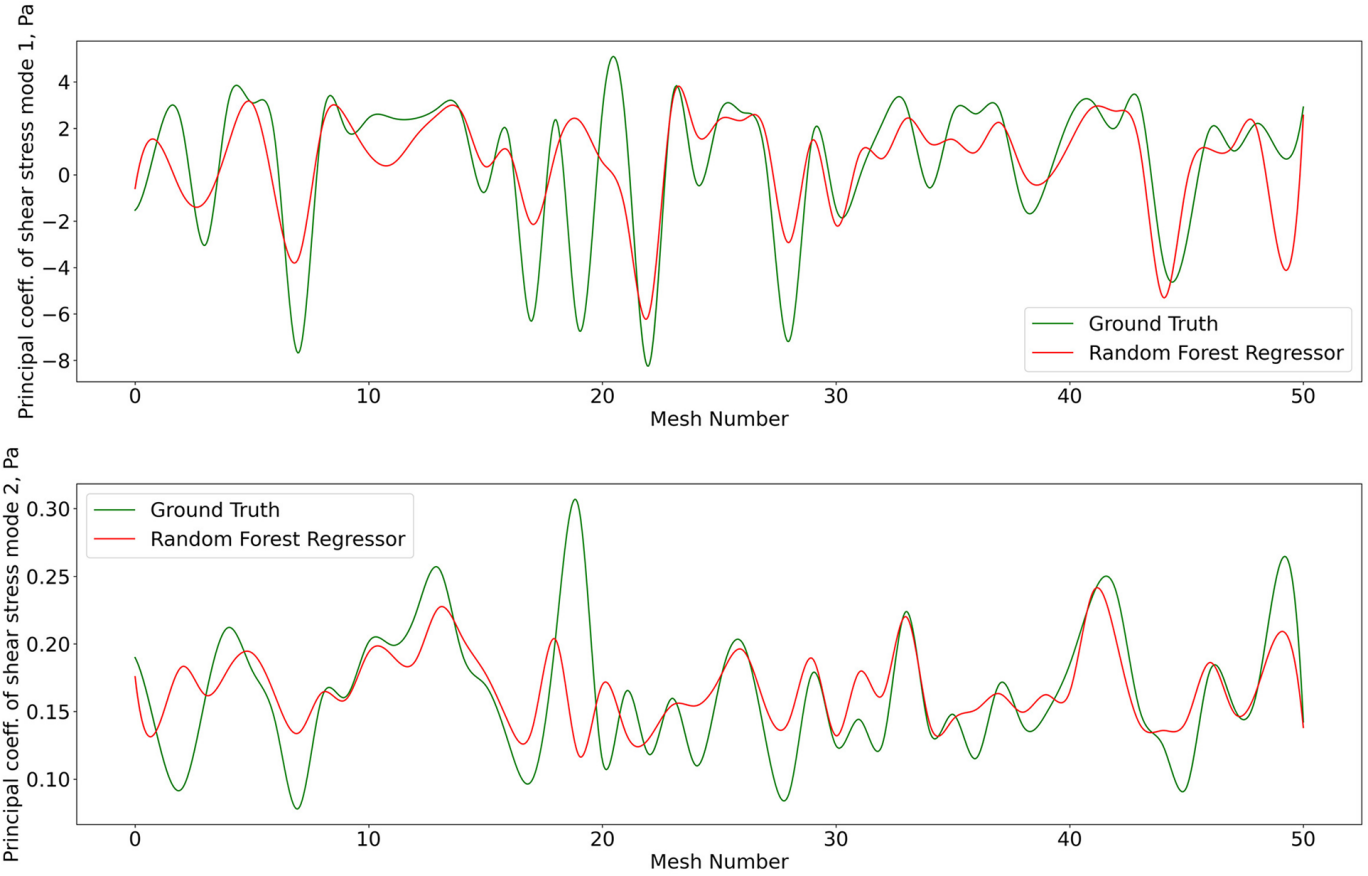
Predictions of POD principal coefficients of shear stress for first two modes using the proposed framework, compared to the ground truth for the test data set. The first part of the same data set was used for training via the RFR. The regression was performed on the 2D t-SNE representation of the meshes against the principal coefficients.

**Figure 9:**
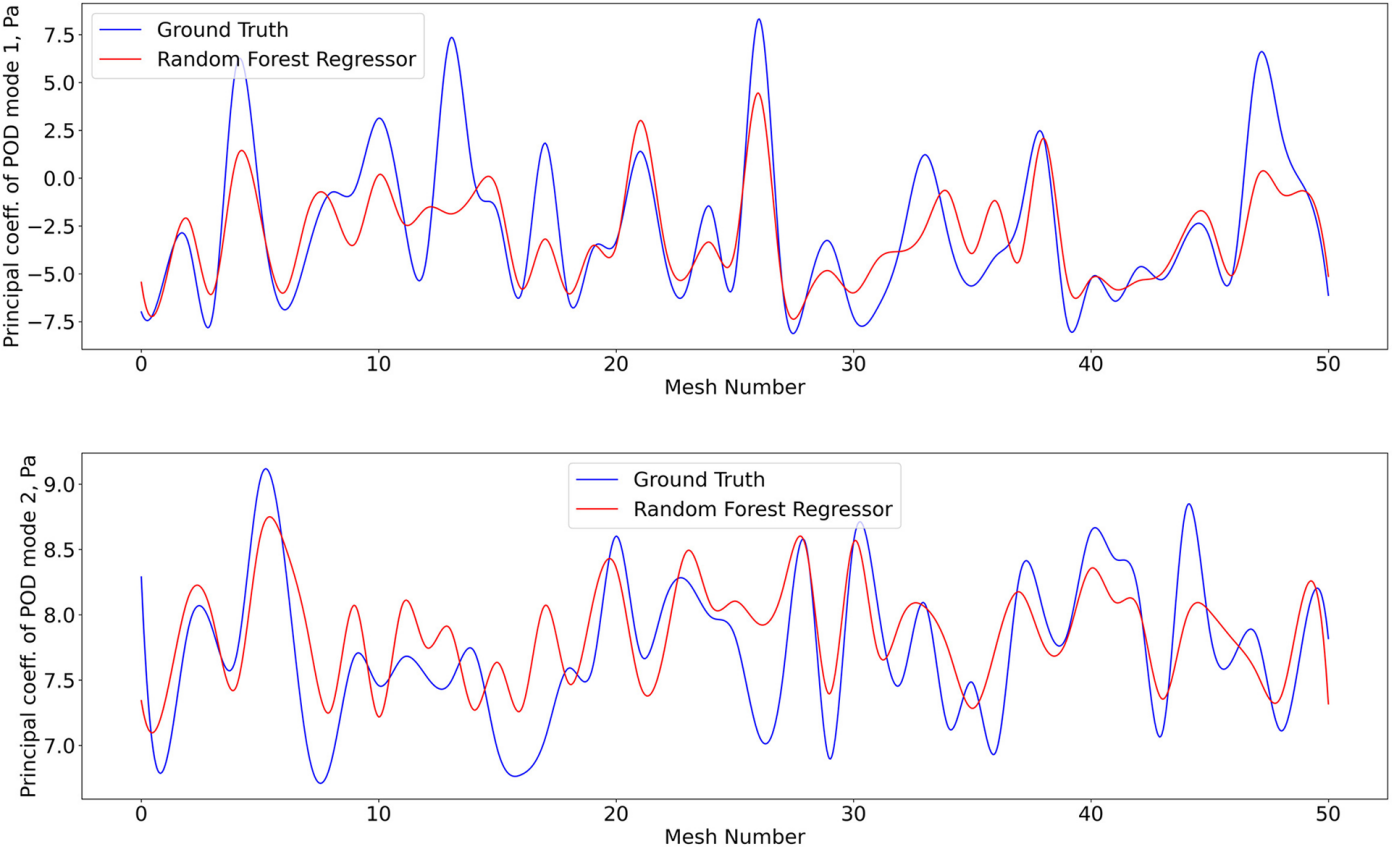
Predictions of POD principal coefficients of pressure for first two modes using the proposed framework, compared to the ground truth for the test data set. Training and testing of the RFR model for pressure utiised the same algorithm, configuration, and optimization as shear stress.

The 1407 t-SNE data points with their respective pressure and shear stress modes were shuffled and divided into a training data set (80% of the overall data) and a validation data set (remaining 20%). The training dataset was used for ten iterations to train the RFR model, where the best maximum tree depth was found to be 20, and the best maximum number of trees for the model was found to be 70. The machine learning model was applied for the test data set as well. Figures 8 and 9 show the results for shear stress and pressure for the two most significant POD modes respectively. The mean Root Mean Square Error (RMSE) of the prediction of the dominant mode coefficient was 15.2% for pressure and 19.7% for shear stress.

**Figure 10:**
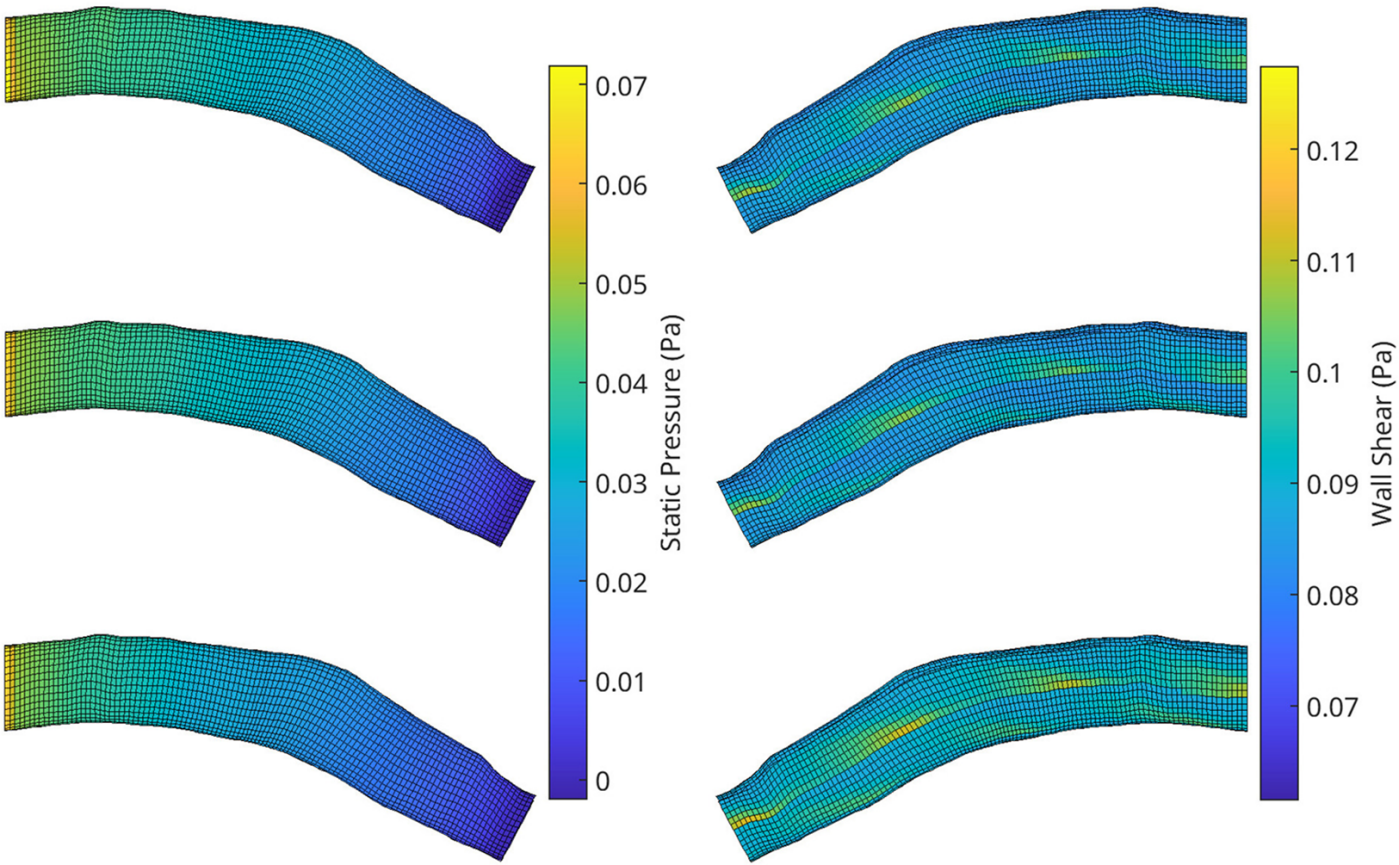
A visualisation of the flow field solution for pressure (left) and wall shear (right) of two test meshes. Shown is the ground truth CFD simulation data (top), the reconstructed POD solution using the 10 most dominant coefficients calculated from the CFD solution (middle) and the reconstruction using the RFR predicted coefficients (bottom).

With the regression for cPOD principal coefficients completed, the mesh-wise modes previously generated by the cPOD method together with the newly predicted coefficients are used to reconstruct the flow field. Results of the 3D reconstruction of the shear stress and pressure fields for the CFD method (“ground truth”) the cPOD reconstruction alone, and the RFR prediction are shown in Figure 10. These were used for further error quantification of the flow solution in the physical space, relative $L_{1}$ and $L_{2}$ norm errors, which are analogues to the normalised mean absolute errors (NMAE) and normalised root mean square errors (NRMSE), respectively, considered in other studies (20). The errors were calculated using the dominant 10 POD modes for the test dataset of 20% of the meshes in accordance with the following definitions:

$$\begin{array}{rl} \mathrm{NMAE}(i=1,\ldots ,\text{imeshmax}) & =\frac{\sum_{\;j=1}^{\;j=\text{\textbackslash{},jnodemax}}|f_{ij}^{\text{ML}}-f_{ij}^{\text{GT}}|}{\text{\textbackslash{},jnodemax}\cdot (\max(f)-\min(f))}\cdot 100\%\\ \mathrm{NRMSE}(i=1,\ldots ,\text{imeshmax}) & =\frac{\sqrt{\sum_{\;j=1}^{\;j=\text{\textbackslash{},jnodemax}}(f_{ij}^{\text{ML}}-f_{ij}^{\text{GT}}{)}^{2}}}{\text{\textbackslash{},jnodemax}\cdot (\max(f)-\min(f))}\cdot 100\% \end{array}$$

where jnodemax is the total number of CFD data points in the considered volumetric/surface distributions, imeshmax is the number of meshes in the test dataset, ML and GT denote the machine learning and the ground truth (CFD) solutions respectively, and f stands for the pressure or wall shear stress solution component. The mean values and the corresponding standard deviations of computed errors are summarised in Table 2. It should be noted that the range of NMAE and NRMSE for pressure is within the accuracy reported for the machine learning models of pressure in aortic flows based on autoencoders and Deep Neural Networks (DNNs) (20). It can also be noticed that the standard deviation and the mean error values are of the same order of magnitude in all cases, which suggests that the populated parameter space for the considered coronary artery problem is relatively sparse. The latter is in agreement with sparsity of the t-SNE maps (Figures 6 and 7). The error variation is particularly large for the shear stresses, which can be explained by a much smaller statistical ensemble of the wall shear surface points in comparison with the volume points where pressure was computed. This is supported by an estimate based on the central limit theorem (21), which suggests that the ratio of statistical errors of the pressure and wall shear stresses should scale as a square root of the ratio of the number of surface points to that of the volume points, and which is about 1:4.5 for all considered meshes.

**Table 2:** Mean errors and standard deviations of reconstructed pressure solution (Left), and of the reconstructed shear stress solution (Right). All values are normalized against corresponding range of values in the full dataset.

NMAE, %	NRMSE, %	NMAE, %	NRMSE, %
2.96±2.84	3.51±3.19	11.21±11.81	11.23±11.81

## 8. Discussion

Rheological theories of Atherosclerosis have been shown to successfully predict plaque location, plaque progression, and plaque rupture (22). They have not been used to infer clinical decisions. Current developments in physics-based artificial intelligence allow us to accelerate these methods so that clinical interventions in the cath lab can be evaluated on novel parameters such as shear stress, pressure drop, and/or velocity field. The main findings of the current paper are that a) synthetic perturbation is an effective way to generate additional surrogate data, which can help satisfy the large volumes required by AI algorithms, b) cPOD, a time-independent variation of POD, can be used to substantially reduce the dimensions of pressure and shear stress field data in simulated blood vessels, c) metrics for quantifying the shape of a blood vessel mesh, such as t-SNE, are effective schemes to drastically reduce the degrees of freedom corresponding to variations in vessel geometry, and d) an interpolative method based on a RFR model was able to predict new pressure fields within seconds, with mean relative $L_{1}$ and $L_{2}$ errors (NMAE and NRMSE) of 2.96% and 3.51% respectively. The errors of the wall shear stress reconstruction show an approximately 4 times larger scatter in comparison with the pressure calculation, in statistical agreement with the smaller number of mesh surface points in comparison with the volume points.

Synthetic manipulations have recently been introduced to Machine Learning to overcome the excessive requirement of well annotated data for AI algorithms (13). We have developed a hybrid approach which took into account the natural variation between blood vessels and applied random synthetic perturbations to produce variants of this original data, with the aim of populating the t-SNE feature space (Figure 2). It was noted that full feature space homogenisation would require significantly more drastic and exotic synthetic manipulation of the OCT data, which would likely negatively impact the ability of the data to represent reality. A better balance between number of real data versus synthetic data is required to bring this technique closer to real-world application. In future, a systematic procedure can be adapted to generate the synthetic meshes in an optimal way by exploiting sensitivity of the coronary flow response to perturbations of the baseline vessel geometry, similar to the deformation matrix method recently developed for aortic flow simulations (23).

Dimensionality reduction helps retain defining features whilst drastically reducing the volume of data required to represent them. This makes machine learning algorithms more likely to identify such features, along with being more computationally efficient. Additionally, it aids in removing noise and extraneous features which can confound important signals (24). In many bio-mechanical applications, autoencoders in combination with DNNs have been a very popular technique to reduce the geometrical complexity to a small set of scalars, which be learnt from the training data. Depending on the DNN calibration, such approaches can be tuned to reproduce the ground truth CFD solution within a few percent relative error (20). However, it can be argued that performance of such methods is strongly dependent on the choice of DNN parameters, while the optimal choice of the latter is application dependent. Differently to the mainstream approach, our method is based on the generalisation of proper orthogonal decomposition (cPOD). This allows for treatment of multiple vectors of the solution matrix of interest simultaneously, which is largely analogous with multiple unsteady flow experiments in fluid mechanics. An important advantage of the POD framework is that it sorts the individual modes in terms of correlative significance. In the current coronary flow simulations, we have considered mesh shape variability as an evolutionary factor for each steady solution component of interest. This is similar to the recent application of Principle Component Analysis (PCA) to data-driven modelling of aortic flows (23), where separate DNN models were used for pressure and absolute velocity. However, in comparison to the standard PCA and DNN techniques, the suggested cPOD approach allows for extension of the solution matrix from single scalars to 3D velocity vectors and pressure components simultaneously on different meshes in space and time.

In unsteady fluid mechanics problems on a fixed mesh, a 1D time coordinate is typically used as an evolutionary variable to characterise the snapshots of the POD method. Here, this approach is generalised to a set of 2D t-SNE coordinates, which are cognate with time for the purpose of POD snapshots and were found sufficient to reconstruct the pressure and wall shear stress fields in any specified blood vessel shape. The t-SNE technique was applied to reduce the complexity of each mesh whilst preserving their characteristic features. In doing so, their relative similarity necessarily remains intact (25) due to the fact that, prior to the embedding step, t-SNE computes the difference between the input meshes based on Euclidean distance between the node coordinates. Therefore, the clustering of the variable phantom meshes around their respective reference shapes arises naturally. Notably, the entire process of meshing the OCT contour domain, embedding this geometry in 2D t-SNE space, predicting the coefficients and constructing the pressure and wall shear stress fields cumulatively takes no more than 2 min, which underpins the success of this method. Furthermore, the applicability of 2D t-SNE coordinates to describe ∼100,000 degrees of freedom corresponding to the number of CFD mesh elements implies a factor of $10^{5}$ dimensionality reduction. In the future, to model multiple solution components in space and time, use of a higher dimensional t-SNE space instead of 2D t-SNE may be reconsidered, and the relationship between clustering accuracy and data sparsity will be investigated.

The standard RFR algorithm was found to be a suitable option for non-linear regression to reconstruct the POD signals from the t-SNE space. Despite the simplicity of the RFR model, the accuracy of predictions was encouraging. Essentially, the model uses the calculated t-SNE co-ordinates and their associated principal coefficients to interpolate the coefficient values over the whole embedding space. The RFR segregates feature data into groups before interpolating within each group, which is particularly suitable for the clustered t-SNE features. Notably, the distribution of mode coefficients in the t-SNE space (Figures 6 and 7) demonstrates smooth variations due to the inherent correlation between the shape of a mesh and the major flow patterns captured by the dominant POD modes.

## 9. Limitations of the method and conclusion

To translate the current method to clinical applications, several limitations must be addressed. First, the current implementation assumes that shape variations are the most important factor affecting velocity fields and their derived parameters. This is corroborated by theoretical arguments, as well as observations that velocity, shear stress and pressure drop strongly scale with diameter. However, the artery flow field also scales with the inflow velocity, which changes throughout the cardiac cycle. To systematically account for the unsteady velocity variation, future developments include extending the scope of the AI model by re-adding the time evolution input. In the meantime, the current simplified steady model may already be sufficient if the flow features of interest are slow compared to the viscous effects, i.e. the flow in the coronary vessel is quasi-steady. In this case, the time history of inflow velocity variation can be decoupled into a series of time frames, where each frame may be represented by a steady process at a different inlet velocity scale. In turn, the shear stress and pressure fields at each frame can be rapidly reconstructed from the inflow velocity and the shear stress and pressure fields of a baseline dataset using the scaling law introduced by Taylor et al. (26).

A more serious limitation of the current study is the neglect of the natural flexibility and heterogeneity of vessel walls in the flow modelling process. Whilst the rigid wall assumption significantly accelerates the solution of the governing Navier-Stokes equations, modelling of the Fluid Structure Interaction (FSI) is essential to correctly capture the coronary artery flow behaviour (27). Hence, future developments will incorporate the FSI model into the simulation driven dataset of the suggested cPOD-tSNE framework.

Despite the overall salutary results of the RFR method, to further refine accuracy of the machine learning model predictions in future, the RFR algorithm may be replaced by more advanced methods such as those based on Gaussian processes; one advantage of which being uncertainty quantification to provide an overall error estimate for the user. Such estimations would be an invaluable addition to a model that is intended for use as a diagnostic tool for clinicians.

Finally, in line with many recent works devoted to the proof-of-concept data-driven modelling of cardio-vascular flows (20), we simplified the model by considering the vessel without side branches. However, it is known that bifurcations occur in the main stem of the left coronary artery, which might affect the inflow conditions. Hence, to reduce the effect of the bifurcation in the current study, the starting site of the 7 catheterised segments was deliberately located 5 vessel diameters downstream of the main stem. Nevertheless, to account for general topology of coronary vessels, which may be of practical interest, the suggested reduced order modelling approach will be extended to side branches in future work.

Despite the above-mentioned limitations of the current work, it can be concluded, using t-SNE and cPOD to perform interpolation by Machine Learning was very successful for the proof-of-concept modelling of coronary artery flows. The speed and accuracy obtained were highly motivating and were able to calculate the pressure and shear stress fields of an unknown vessel within seconds. Rheological theories of Atherosclerosis have been shown to successfully predict plaque location, plaque progression, and plaque rupture (22), but they have not been used to infer clinical decisions. Current developments in physics-based AI allow us to accelerate these methods such that clinical interventions in the cath lab can be evaluated on novel parameters such as shear stress, pressure drop and 3D velocity field.

To conclude, we developed a method to produce a very fast solution to the Navier-Stokes equations, as we aimed to focus on applying this method in a clinical environment with high demand for rapid solutions. We are currently working towards newer methods enabling time dependent flows that incorporate solid state interactions, as well as higher accuracy AI modelling functions with corresponding error estimates.

## Data Availability

The raw data supporting the conclusions of this article will be made available by the authors, without undue reservation.
